# Progressive cracking technique for phacoemulsification of superhard cataracts: a case report

**DOI:** 10.1186/s40662-019-0163-0

**Published:** 2019-12-01

**Authors:** Yun-e Zhao, Zhangliang Li, Pingjun Chang, Dandan Wang, Man Hu

**Affiliations:** 1 School of Optometry and Ophthalmology and Eye Hospital, Wenzhou Medical University, 270 West Xueyuan Road, Wenzhou, 325027 Zhejiang China; 2 Key Laboratory of Vision Science, Ministry of Health People’s Republic of China, Wenzhou, Zhejiang China

**Keywords:** Cataract, Surgical technique, Phacoemulsification

## Abstract

**Background:**

Complete nuclear disassembly of superhard cataracts cannot always be achieved by phaco chop, which is considered one of the best techniques for dealing with hard cataracts. We present a phaco chop-progressive cracking technique to divide superhard cataracts completely.

**Case presentation:**

We presented a case of cataract with over Grade V nucleus sclerosis and very low density of corneal endothelial cell (812 cells/mm^2^). By performing the cataract surgery with our phaco chop-progressive cracking technique, the corneal endothelial cells were well protected and the patient’s visual acuity was markedly improved from finger counting at 40 cm to 20/200 the day after surgery without obvious corneal edema.

**Conclusions:**

Although an initial learning curve was needed, this phaco chop-progressive cracking technique could be of particular benefit to the superhard cataract, especially in patients with low density of corneal endothelial cells.

## Background

Phacoemulsification with intraocular lens implantation has become the first line choice for most ophthalmologists in cataract removal procedures. However, phacoemulsification of superhard cataracts remains a challenge even to experienced surgeons due to the extra maneuver and energy needed in such cases. Excessive cell loss in corneal endothelium is of great concern [1]. The integrity of the rhexis, posterior capsule, and zonules are often at high risk of rupture. Many surgeons choose manual extracapsular cataract extraction (ECCE) for superhard brunescent or black cataracts. Small-incision cataract surgery (SICS), also called manual small-incision cataract surgery (MSICS), is also a safe and cost-effective procedure for dense cataracts [2]. However, faster visual recovery and lower risk of subchoroidal expulsive hemorrhage make endocapsular phacoemulsification advantageous over manual cataract surgery, especially in monocular cases.

The biggest challenge for the surgeon in superhard cataract is to completely divide the nucleus without impairing the other intraocular tissues. The phaco-chop technique introduced by Nagahara K et al. has become popular in dense cataract management, due to its high efficiency and little stress on capsule bag and zonules [3]. It enables appropriate and safe division of hard nucleus in most cases. In superhard cataracts with leathery posterior plates, however, the regular phaco chop technique often proves inadequate. Despite various modifications of the phaco chop technique [3] and divide-and-conquer technique [4], high chances of intraoperative complications still hinder many surgeons from performing phacoemulsification in superhard cataracts.

The progressive cracking technique presented here is a safe, effective and efficient way to disassemble dense and even leathery cataracts completely with minimal risk to the anterior and/or posterior capsule, zonules and endothelium.

## Case presentation

A 76-year-old female came to our clinic in January 2016 complaining of vision deterioration in both eyes for 2 years. The corrected distance visual acuity (CDVA) was finger counting at 40 cm in the right eye and 20/200 in the left eye, which presented over Grade V nucleus sclerosis in both eyes [5]. The axial length was 29.60 mm in the right eye and 30.70 mm in the left eye. The visual acuity measured by a Retinal Acuity Meter (RAM) was 20/200 in the right eye. The corneal endothelial cell density was 812 cells/mm^2^ in the right eye and 748 cells/mm^2^ in the left eye counted automatically, using noncontact specular microscopy (KONAN).

Cataract surgery was performed in the right eye. Ophthalmic viscosurgical device (OVD) was used to protect corneal endothelium [6]. The OVD (VISCOAT: sodium chondroitin sulfate 40 mg/ml + sodium hyaluronate 30 mg/ml, and PROVISC: sodium hyaluronate 10 mg/ml, Alcon Laboratories, Inc.) was injected into the anterior chamber. Viscoat was injected before Provisc. The hard nucleus was divided by the progressive cracking technique and emulsified, followed by irrigation/aspiration and a single-piece Akeros MI60 intraocular lens (IOL) (Bausch & Lomb, Inc.) was implanted uneventfully.

### Surgical technique

Surgery starts with a 2.2 mm clear corneal incision under topical anesthesia at 11 o’clock. A side port is created at 3 o’clock, followed by a continuous curvilinear capsularhexis (CCC) and hydrodissection to achieve complete cleavage of cortex from the capsule. The size of the CCC is between 5.5 mm to 6.0 mm. The phaco probe is introduced into anterior chamber through the clear corneal incision. We prefer having the phaco tip bevel down facing the nucleus.

**Fig. 1:**
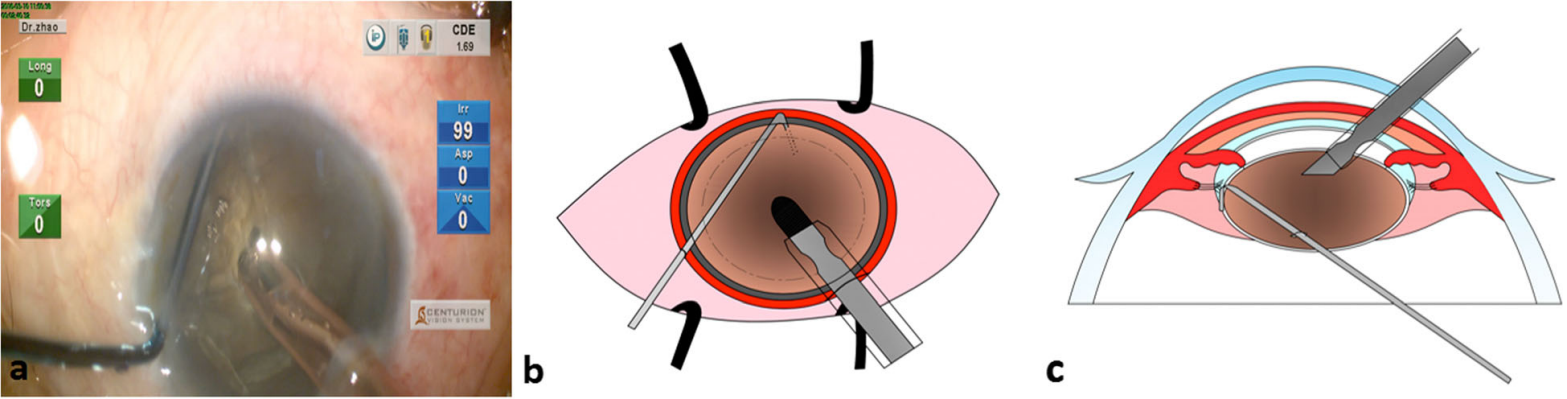
Schematic diagram of step 1. (**a**) A screenshot to show the procedure that the entry point for a phaco tip is slightly axial to the rhexis and the phaco tip extended into and towards the very center of the nucleus with preset maximal energy. (**b** & **c**) Drawings of front side and lateral side

Step 1: The phaco tip was embedded into the nucleus with preset maximal energy. The entering site of the phaco tip was slightly axial to the rhexis, so the end of the tip will reach the very center of the nucleus, which is usually considered the densest part. It was important to have sufficient exposure of the phaco tip from the sleeve. In our experience, the phaco tip is usually exposed about 1 mm for dense cataracts. Complete burying of the exposed phaco tip enabled firm holdability of the nucleus, which is crucial for the following chopping. Given the thickness of the dense cataract, this maneuver was perfectly safe (Fig. 1).

**Fig. 2:**
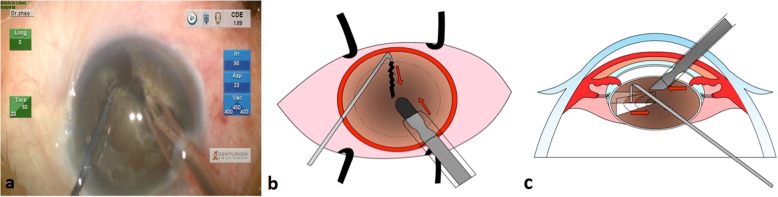
Schematic diagram of step 2. (**a**) A screenshot to show the procedure that an initial crack was created when the chopper was pulled towards the phaco tip with the phaco tip holding the nucleus. (**b** & **c**) Drawings of front and lateral side, respectively

Step 2: The chopper was introduced through the side port, parallel to the anterior capsule, and placed close to equator underneath the rhexis. With the phaco tip holding the nucleus, the chopper was pulled towards the phaco tip to create an initial crack (Fig. 2).

Step 3: It is important to realize that a complete chop is not mandatory. With an incomplete chop of the nucleus in the inferior part of the nucleus, the nucleus presents left and right halves, usually with the superior parts still connected. The phaco tip was introduced into the right hemisphere from the wall of the crack. Once the vacuum reaches the maximal setting, which meant that the stable holding force is available, the surgeon slightly lifted the nucleus anteriorly. This ensured that the next part of procedure took place away from the posterior capsule, especially in cases when there is no cortex to act as cushion around the nucleus. The surgeon moved the chopper from distal to proximal along the existing crack, and extended the crack till it was complete across the whole lens. This was a progressive cracking process, which showed no overly aggressive movements. In most superhard cataract cases, the subincisional parts of the two halves may still be connected. The surgeon will then rotate the nucleus 180 degrees, and repeat the horizontal phaco chop-progressive cracking till a complete chop and crack was made.

**Fig. 3:**
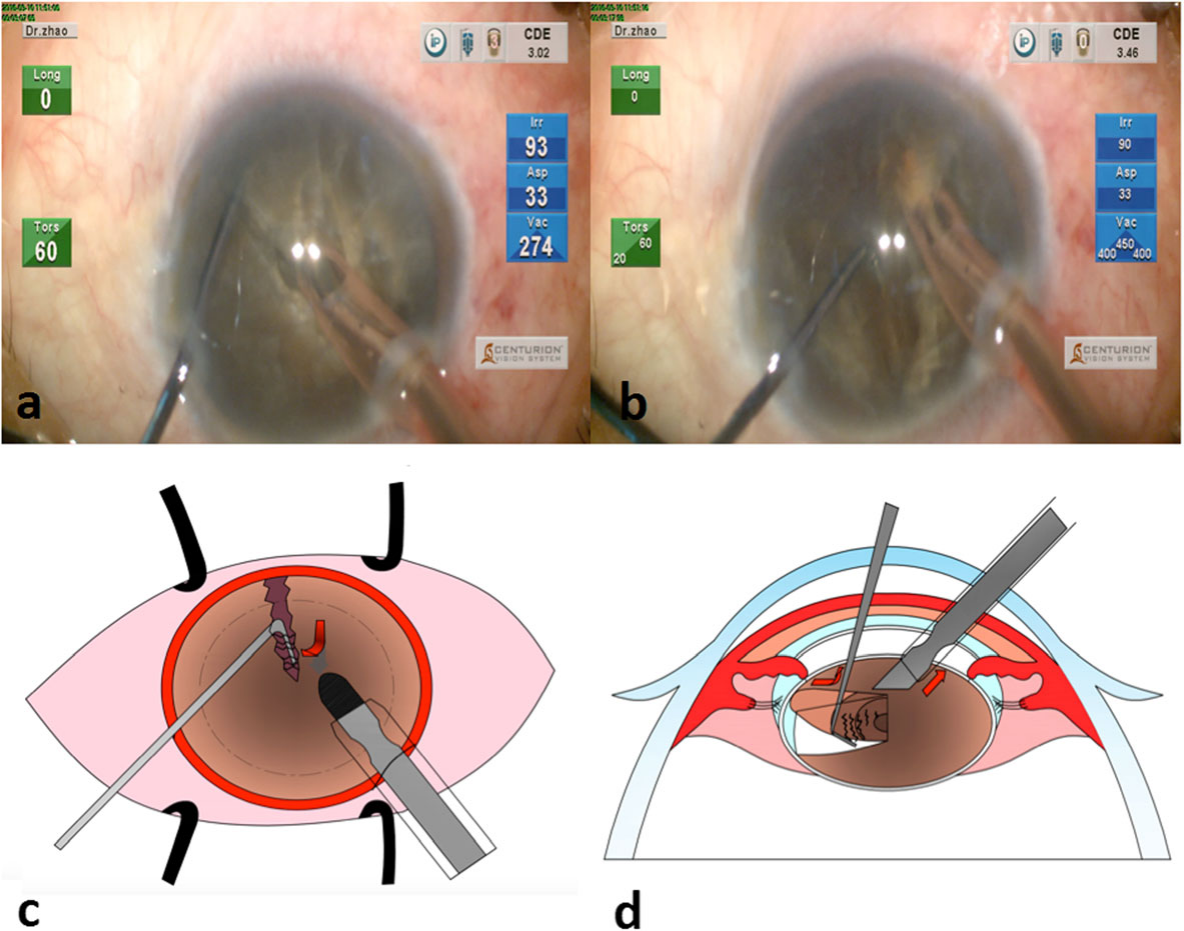
Schematic diagram of step 3. Once the vacuum reaches the maximal setting, the surgeon slightly lifts the nucleus anteriorly (**a**) The surgeon moved the chopper from the distal position to the proximal position along the existing crack, and extended the crack till it was complete across the whole lens. In super dense lens, the surgeon placed the chopper at the bottom of the crack, and cut across the strands upwards (**b**, **c** and **d**)

In the super dense lens, the posterior leathery strands connecting the two halves are common. In such circumstances, the surgeon injects some dispersive OVD underneath the nucleus and placed the chopper at the bottom of the crack, and cut across the strands upwards (Fig. 3a, b, c & d).

Once the lens was divided into two halves, the surgeon continued the phaco chop to separate the big fragments into smaller fragments like in regular cases. When needed, we employed the same strategy, phaco chop- progressive cracking in the disassembly.

**Fig. 4:**
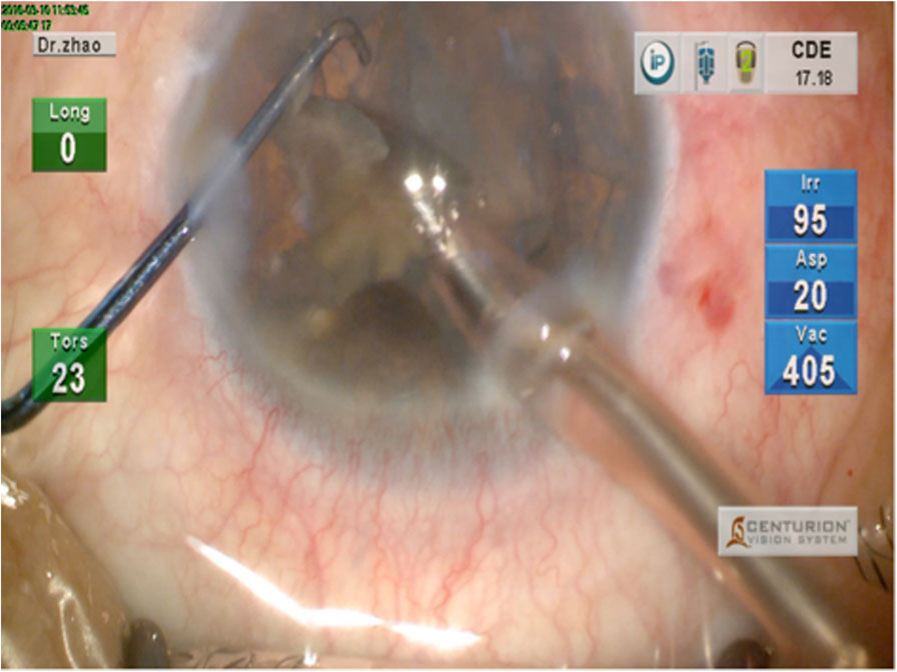
Schematic diagram of step 4. When emulsifying the wedge-shaped fragments, the surgeon kept the bevel of phaco tip facing the sharp apex, using the chopper to stabilize the fragment

Step 4: When emulsifying the wedge-shaped fragments, the surgeon kept the bevel of phaco tip facing the sharp apex, using the chopper to stabilize the fragment. This prevented the sharp edges from rupturing the capsule (Fig. 4).

The Alcon Centurion Vision System was used with the preset parameters as follow: Torsional ultrasound energy 30–60%, vacuum of 450 mmHg (with intelligent phaco setting: when vacuum approaches 450 mmHg, longitudinal ultrasound initiates), aspiration flow rate of 45 cc/min, bottle height of 90 cm from the patient’s eye level, cumulative dissipated energy (CDE) of 21.70, total ultrasonic time of 89 s, estimated fluid usage of 67 ml.

The CDVA was improved to 20/200 in the right eye the second day after surgery. No corneal edema was seen. The noncontact specular microscopy reported 1021 endothelial cells per square millimeter in the right eye 1 week after surgery. This increased density of endothelial cells might due to the artifact that different regions were measured.

## Discussion and conclusions

Dense cataracts often present with a stubborn leathery posterior plate, which leads to excessive maneuver, prolonged surgical time, and increased phaco energy consumption as well as more fluids getting in and out of the eye. Many causes are believed to be related with the endothelial cell loss [1]. The phaco energy used, the irrigation of the fluids, the tumbling of the small pieces of lens materials, and the osmotic features of the irrigation balanced salt solution are all considered relevant. An effective, efficient and complete separation of the bulky dense cataract into smaller fragments is of great importance to reduce phaco energy consumption, surgical time and irrigation volume. In this paper, we introduce a new technique to achieve complete, safe, effective and efficient nucleus disassembly.

We use phaco chop to create the initial crack of the lens, then employ progressive cracking to completely separate the nucleus. Compared with divide-and-conquer and stop-and chop, there was less energy dissipated with phaco chop technique. However in superhard cataract, phaco chop alone was often insufficient to achieve complete disassembly. With progressive cracking, the surgeon used the phaco tip to stabilize one half of the lens from the initial chop and used a chopper to extend the crack progressively. There was only minimal stress on the capsule or the zonules as there was no aggressive movement of the instruments.

With regular phaco chop technique, the leathery plate often becomes leathery strands, keeping the hemispheres connected posteriorly. In such circumstances, the surgeon placed the chopper at the bottom of the crack without touching posterior capsule, and moved the chopper forward to cut across the strands. Kamoi et al [7] reported a forward-chop technique to manage the leathery plate. In the forward-chop technique, the surgeon attempted phaco chop first, resulting in incomplete separation due to the characteristics of the nucleus. The surgeon then dislocated the right hemisphere anteriorly till it was 50% above the rhexis plane, then a chopper was placed behind the right hemisphere followed by chopping anteriorly with the phaco tip holding the lens. With forward chop, the surgeon needed to perform a large rhexis (around 6 mm in diameter) to ensure the anterior dislocation of the lens. It was different from the forward-chop technique that we hold the lens with maximal vacuum, when the phaco tip was embedded into the right hemisphere, and divided the nucleus without anterior dislocation of the lens.

Vasavada et al reported a multilevel chop technique for phacoemulsification in dense cataracts [8]. With the multilevel chop technique, the surgeon achieved complete separation by repositioning the chopper as well as the site of occlusion with the probe at multiple planes. It can be used in both horizontal and vertical chop techniques. In our technique, we did not repeat embedding the phaco tip in multiple levels. The progressive cracking and multilevel chop share one common feature, which is that the surgeon can achieve complete separation of dense nucleus by a progressive approach without delivering too much stress on the capsule or zonules. This is particularly important in eyes that are more susceptible to capsular rupture or zonular dehiscence.

Thanks to Kamoi et al*’s* forward-chop technique [7] and Vasavada *et al’s* multilevel chop technique [8], we built and improved our progressive cracking technique inspired by their techniques.

It is also important to use high quality dispersive OVD to coat and protect the endothelium. The surgeon reapplied the dispersive OVD when the surgical time was long and the OVD coated on the endothelium might be removed due to long time irrigation. The dispersive OVD was also used as cushion around the nucleus in hypermature cataract cases, in which the cortex was liquefied while the lens was very dense. In this case, the patient had a very low density of corneal endothelial cells and dense cataract, so we employed dispersive OVD and progressive cracking technique to protect the endothelium. As a result, it is an effective way to delay endothelial keratoplasty, which is meaningful to countries with shortage of donated corneas. The patient’s postoperative visual acuity was not good because of retinal atrophy caused by pathologic myopia.

The following should be considered carefully when employing the progressive cracking technique [6]. A thorough hydrodissection is important in this technique. Cortical cleaving hydrodissection in multiple quadrants may make rotation easier [9]. However in cases with weak zonules, the surgeon needs to be very gentle when rotating the lens. Artificial cushion with a quality dispersive OVD helps to protect capsular bag, especially the posterior capsule, when managing hypermature dense cataract that has no or very little cortex, or in the cases where the cortex is liquefied.

In conclusion, the progressive cracking technique might reduce the risk of many common intraoperative complications such as endothelial cell loss, posterior capsule rupture and zonular dialysis. Despite there being a learning curve in the beginning, it provides safe, effective and efficient phacoemulsification in patients with superhard cataracts.


**Additional file 1:** **Video S1.** Progressive cracking technique in a superhard cataract extraction.


## Data Availability

All data generated or analyzed during this study are included in this published article [and its supplementary information files].

## References

[1] Storr-Paulsen A, Norregaard JC, Ahmed S, Storr-Paulsen T, Pedersen TH (2008). Endothelial cell damage after cataract surgery: divide-and-conquer versus phaco-chop technique. J Cataract Refract Surg.

[2] Dean WH (2015). Quality of small incision cataract surgery. Community Eye Health.

[3] Nagahara K (1993). Phaco-chop technique eliminates central sculpting and allows faster, safer phaco. Ocular Surgical News.

[4] Gimbel HV (1991). Divide and conquer nucleofractis phacoemulsification: development and variations. J Cataract Refract Surg.

[5] Chylack Leo T. (1993). The Lens Opacities Classification System III. Archives of Ophthalmology.

[6] Storr-Paulsen A, Norregaard JC, Farik G, Tårnhøj J (2007). The influence of viscoelastic substances on the corneal endothelial cell population during cataract surgery: a prospective study of cohesive and dispersive viscoelastics. Acta Ophthalmol Scand.

[7] Kamoi K, Mochizuki M (2010). Phaco forward-chop technique for managing posterior nuclear plate of hard cataract. J Cataract Refract Surg.

[8] Vasavada AR, Raj SM (2011). Multilevel chop technique. J Cataract Refract Surg.

[9] Fine IH (1992). Cortical cleaving hydrodissection. J Cataract Refract Surg.

